# Study of Materia Medica

**Published:** 1836-01

**Authors:** 


### STUDY OF MATERIA MEDICA.

It is agreeable to reflect on the increased and increasing opportunities afforded to students of physic, of making themselves familiar with the drugs they will afterward have daily to prescribe. A museum of Materia Medica has been recently established by the Royal College of Physicians, Edinburgh; and the object, as stated in the Edinburgh Medical and Surgical Journal, is to have a complete collection both of the ordinary and more rare articles employed in medicine. The College invite professional men and naturalists in the colonies, and other favorable situations, to add to their specimens; and we hope the invitation will be attended to. Looking back some twenty years, one can hardly now believe that students of old might pass through their curriculum, and take a medical degree, without having ever seen any of the articles of the materia medica, and without knowing one article from another. This is certainly one of the improvements of late years. We presume the museum in the Edinburgh College of Physicians will be accessible to teachers and students, without which the collection will do little more than serve the purpose of idle display.

